# Why are Some Games More Addictive than Others: The Effects of Timing and Payoff on Perseverance in a Slot Machine Game

**DOI:** 10.3389/fpsyg.2016.00046

**Published:** 2016-02-02

**Authors:** Richard J. E. James, Claire O’Malley, Richard J. Tunney

**Affiliations:** ^1^School of Psychology, University of NottinghamNottingham, UK; ^2^School of Psychology, University of Nottingham, Malaysia CampusSemenyih, Malaysia

**Keywords:** gambling, impulsivity, associative learning, behavior, addictive, reinforcement schedule, slot machine

## Abstract

Manipulating different behavioral characteristics of gambling games can potentially affect the extent to which individuals persevere at gambling, and their transition to problematic behaviors. This has potential impact for mobile gambling technologies and responsible gambling interventions. Two laboratory models pertinent to this are the partial reinforcement extinction effect (PREE) and the trial spacing effect. Both of these might speed up or delay the acquisition and extinction of conditioned behavior. We report an experiment that manipulated the rate of reinforcement and inter trial interval (ITI) on a simulated slot machine where participants were given the choice between gambling and skipping on each trial, before perseverative gambling was measured in extinction, followed by measurements of the illusion of control, depression and impulsivity. We hypothesized that longer ITI’s in conjunction with the low rates of reinforcement observed in gambling would lead to greater perseverance. We further hypothesized, given that timing is known to be important in displaying illusory control and potentially in persevering in gambling, that prior exposure to longer intervals might affect illusions of control. An interaction between ITI and rate of reinforcement was observed, as low reinforced gamblers with a long ITI gambled for longer. Respondents also displayed extinction and a PREE. Gamblers exposed to a higher rate of reinforcement gambled for longer in acquisition. Impulsivity was associated with extended perseverance in extinction, and more depressed gamblers in the high reinforcement short ITI group persevered for longer. Performance in the contingency judgment failed to support the second hypothesis: the only significant contrast observed was that participants became better calibrated as the task progressed.

## Introduction

The emergence of new gambling technologies comes with the concern that novel reinforcement schedules might increase the risk of harm to gamblers. Models of problem gambling assume there are a set of common behavioral and cognitive processes underpinning the development of addictive behavior (Blaszczynski and Nower, 2002; Sharpe, 2002). We report an experiment investigating the effects of partial reinforcement and timing on perseverative gambling behavior, as these may underpin part of the transition to problem gambling. Deficits in processing partial reinforcement have been previously observed in heavy gamblers (Horsley et al., 2012), while increasing inter trial intervals (ITIs) facilitates the acquisition of conditioned behavior (Gallistel and Gibbon, 2000). In this report we outline an experiment in which participants played on a simulated slot machine on which win frequency and ITI were manipulated between groups and perseverance in extinction was measured.

### Delay, Trial Spacing, and ITIs

Increasing the interval between gambles might be instrumental in encouraging continued play and may be a component behind the popularity of certain games. Lottery games for example have extended delays between gambles and are often the most popular and frequently played games (Wardle et al., 2011). While this might be because lotteries are highly available (amongst numerous considerations), in some jurisdictions (e.g., the UK) other games are offered alongside lottery tickets (e.g., scratchcards), controlling for availability. Despite this, many more people play the lottery than similarly available games, and do so more frequently. However, the perceived risk of harm is very low, although it is unclear whether the ‘addictiveness’ of gambling lies in specific games (Afifi et al., 2014) or specific behavioral features (Griffiths and Auer, 2013). Some mobile video games exploit similar effects by enforcing delays between plays of gambling-like games. In-play betting, which is associated with mobile (Hing et al., 2014) and problem gambling (Gray et al., 2012; LaPlante et al., 2014), combines continuous and discontinuous play. Understanding the role of timing and latency on gambling behavior has important consequences for newer forms of gambling, such as mobile gambling (where in-play betting is heavily promoted), as the manner in which people use smartphones is likely to increase latencies between gambles. In-play refers to bets made on an event (e.g., a soccer match) while the event itself is occurring whereas in traditional forms of betting the wager is made prior to the event. Griffiths and Auer (2013) argue in-play betting might be more addictive because it is more continuous. However, considerable discontinuities persist in play as betting remains constrained within an event. Real data on in-play betting (LaBrie et al., 2007) reveals mixed findings: although there is a clear risk of problem gambling, the findings do not decisively conclude this is because of its continuous nature; in-play gamblers placed fewer bets and there was little difference in daily levels of betting. Although in-play bettors wagered more money overall, the median wagered was lower than traditional sports betting, and in-play bettors had a lower net loss. Gray et al. (2012) suggest the immediacy between wager and outcome may be instrumental in attracting risky or impulsive gamblers to in-play gambling.

The associative learning literature indicates that increased latencies between reinforcements facilitate acquisition of conditioned behaviors (Gallistel and Gibbon, 2000). Gallistel and Gibbon’s (2000) timing model hypothesizes that a decrease in the ratio between reinforcements and ITI in classical and operant conditioning reduces the number of reinforcements to acquisition. This is claimed to be independent of partial reinforcement, which increases the number of *trials* but not *reinforcements*. The literature on the ‘trial spacing’ effect, primarily studied in the context of classical conditioning (Barela, 1999; Stout et al., 2003; Sunsay et al., 2004; Moody et al., 2006; Sunsay and Bouton, 2008; Miguez et al., 2014), has found that dispersed trials facilitate conditioning.

It is less clear whether greater latencies in extinction affect performance. Gallistel and Gibbon (2000) claim that the interval without reinforcement rather than non-reinforcing events is key, and that omitted reinforcements in extinction are unaffected by partial reinforcement. Other research has identified ITI effects on extinction, with greater suppression of responding observed with shorter ITI’s (Mackintosh, 1974; Moody et al., 2006).

Timing is thought to be an important component of the illusion of control (Msetfi et al., 2005, 2007; Baker et al., 2010), a cognitive bias that is prevalent in problem gambling (Fortune and Goodie, 2012). Illusions of control, operationalized as an overestimation of the relationship between a response and outcome, can be induced using a contingency judgment task in which these events are unrelated but the outcome occurs very frequently. Standard examples of this task include a button pushing task associated with the activation of a light (Alloy and Abramson, 1979), or a medical decision-making task judging the relationship between an experimental drug and patient improvement (Orgaz et al., 2013). The extent to which non-depressed individuals show illusions of control is affected by the latency between trials: longer ITI’s are associated with stronger illusory control in non-depressed individuals (Msetfi et al., 2005). Problem gamblers show stronger illusions of control in contingency judgment paradigms (Orgaz et al., 2013), although the causal direction of this relationship remains unclear: extensive exposure to certain schedules of reinforcement might increase illusions of control, or individuals susceptible to illusions of control may be more likely to develop gambling problems. We included a task derived from the same paradigm as Orgaz et al. (2013), which participants were asked to complete after the slot machine task. We also measured depression as depressed individuals appear to make more calibrated judgments in this paradigm (Alloy and Abramson, 1979) with a longer ITI (Msetfi et al., 2005). Disordered mood has also been identified as a potential pathway to problem gambling (Blaszczynski and Nower, 2002).

### Partial Reinforcement Extinction Effect and Impulsivity

The partial reinforcement extinction effect (PREE) is a behavioral paradox in which weakly reinforced behaviours persist for longer without reinforcement relative to more consistently occurring reinforcers (Mackintosh, 1974; Bouton et al., 2014), such as during an extended period of losses in gambling (Dickerson, 1979; Fantino et al., 2005; Horsley et al., 2012). Partial reinforcement deficits have been identified in high frequency gamblers^1^, who take longer than recreational gamblers to extinguish these associations (Horsley et al., 2012), a change that might occur from chronic exposure to the schedules of reinforcement in gambling. Horsley et al. (2012) report that although partial reinforcement is hypothesized to be an important component in gambling, the evidence base is sparse. Failure to extinguish has been identified as a marker of problem gambling (Weatherly et al., 2004). Failure to extinguish also directly (e.g., unsuccessful efforts to stop gambling, gambling more than intended to) or indirectly (e.g., chasing losses) corresponds onto indicators for Gambling Disorder (American Psychiatric Association, 2013) or problem gambling (Lesieur and Blume, 1987).

It is unsurprising that the PREE has been linked with gambling, and considerable attention has been devoted to studying this in slot machines. Slot machines tend to have a very low rate of reinforcement (although this varies on computerized machines), and gamblers persevere in play despite mounting sequences of losses. There is a literature that has used slot machine tasks to probe the effects of partial reinforcement on operant learning. Lewis and Duncan (1956, 1957, 1958a,b) conducted a series of experiments using simulated gambling to test theories of partial reinforcement, finding that lower reward probabilities were associated with greater perseverance. Poon and Halpern (1971) used a similar paradigm to test Capaldi’s (1966; Capaldi and Martins, 2010) partial reinforcement theories by manipulating trial order in a slot machine task with a small number of acquisition trials. Kassinove and Schare (2001) manipulated big wins and near-misses in perseverative behavior in extinction in a similar slot machine paradigm, finding that near-miss density affected the extent to which participants persisted gambling but not big wins.

Different schedules of reinforcement potentially affect how behaviors extinguish (Madden et al., 2007; Haw, 2008a) Gambling operates on a random ratio schedule of reinforcement, a subset of the variable ratio schedule. Less well understood than variable ratio schedules, it is informative to contrast how random ratio schedules differ from variable ratio schedules. The typical distribution the number of trials until a response is reinforced on a random ratio schedules follows an L-shaped pattern; the number of trials rapidly drops off after a small number of plays but continues indefinitely at very low probability. In contrast on a variable ratio schedule it is usually (but not necessarily) the case that the probability of the number of trials to reinforcement is evenly distributed, and there is an upper limit on the number of trials before a behavior is reinforced (Haw, 2008a). Studies comparing these schedules have not shown clear differences; Hurlburt et al. (1980) found no difference between variable and random ratio schedules in gambling, although weaknesses with this study have been identified (Haw, 2008b). Crossman et al. (1987) found no difference between three ratio reinforcement schedules (variable, fixed and random) in animals. Recent studies have suggested that random-ratio schedules demonstrate more perseverative behavior compared to fixed-ratio schedules, particularly when the number of trials to reinforcement is very large (Madden et al., 2007).

The slot machine task we outline in this report was designed so that participants were asked to risk money they had won during the experiment, but the amount of money won would gradually increase. The low-reinforcement conditions attempted to create a situation similar to real-money gambling. One criticism of many slot machine experiments was that these studies tended to utilize a high rate of reinforcement relative to real slot machines (Kassinove and Schare, 2001; Harrigan, 2007). A mechanical three-reel slot machine has a win probability of 9%, but this varies on computerized machines (Harrigan and Dixon, 2009). In gambling research (e.g., MacLin et al., 2007; Dixon et al., 2011) higher rates of reinforcement (20%) have been used in extinction paradigms. We decided to use a rate of reinforcement of 30%, operating on a random ratio schedule of reinforcement similar to real slot machine gambling.

Self-reported impulsivity was measured impulsivity predicts perseverative gambling in the face of mounting losses, and is a pathway to problem gambling. Breen and Zuckerman (1999) found that impulsive gamblers ‘chased’ losses for longer in a gambling game where the win probability decreased as the experiment continued. Impulsivity has been identified as risk factor for problem gambling, problem gamblers (MacLaren et al., 2011; Kräplin et al., 2014) show higher self-reported impulsivity.

To test whether these behavioral effects encourage perseverative gambling, we conducted a two-part experiment where ITI and rate of reinforcement were manipulated. Participants were assigned to one of four groups and exposed to a high or low rate of reinforcement, and a long or short ITI between gambles. Associations were extinguished after a certain amount of money had been won. Participants subsequently completed a contingency judgment task in which they judged the efficacy of an experimental drug. The literature on partial reinforcement predicts that individuals exposed to a lower rate of reinforcement will persevere longer. Trial based accounts of extinction predict that massed extinction trials should suppress responding faster, as opposed to a timing-based account where there ought to be no difference. Impulsive gamblers should persevere for longer in extinction as well, on the basis of previous experiments looking at perseverance in loss-chasing.

## Materials and Methods

### Design

The experiment was a 2 × 2 between-subjects factorial design, the rate of reinforcement and ITI were the factors manipulated. The rates of reinforcement were 0.7 and 0.3. ITIs were either long (10 s) or short (3 s).

On every trial the participants were given the choice either to gamble or not. The number of trials in which participants decided to gamble was the dependent variable. The outcome of the gamble and the amount of money participants had won was also recorded. The extinction phase was divided into blocks of 10 trials for analysis. Participants were also administered a contingency judgment task. In the contingency judgment task measures were of the proportion of trials in which the drug was administered, and the contingency judgment made by participants. Impulsivity and depression were measured using the Barratt Impulsiveness Scale (BIS-11; Patton et al., 1995) and Beck Depression Inventory (BDI; Beck et al., 1961) respectively. The BIS-11 is a 30-item measure that measures three higher order factors of attentional, non-planning, and motor impulsivity (Patton et al., 1995). The BDI is a 24-item measure that measures multiple levels of depression severity, discriminates depression from anxiety and has strong internal consistency (Beck et al., 1988). No further measurements of individual difference or behavior were taken apart from the ones reported herein.

### Participants

A total of 122 participants were recruited from the University of Nottingham community to take part in this study (Mean age = 22.63, *S.D.* = 3.96, gender – 69 females and 53 males)^2^. This study was carried out in accordance of, and with ethical approval by the University of Nottingham School of Psychology Ethics Review Committee. All participants gave written consent prior to the beginning of the experiment.

There was no evidence of any trait differences between the groups. A one-way Analysis of Variance (ANOVA) was conducted on both questionnaires, and the ANOVAs for the BIS [*F*(4,166) = 1.543, *p* = 0.192] and the BDI [*F*(4,166) = 0.662, *p* = 0.619] were non-significant.

### Procedure

**FIGURE 1:**
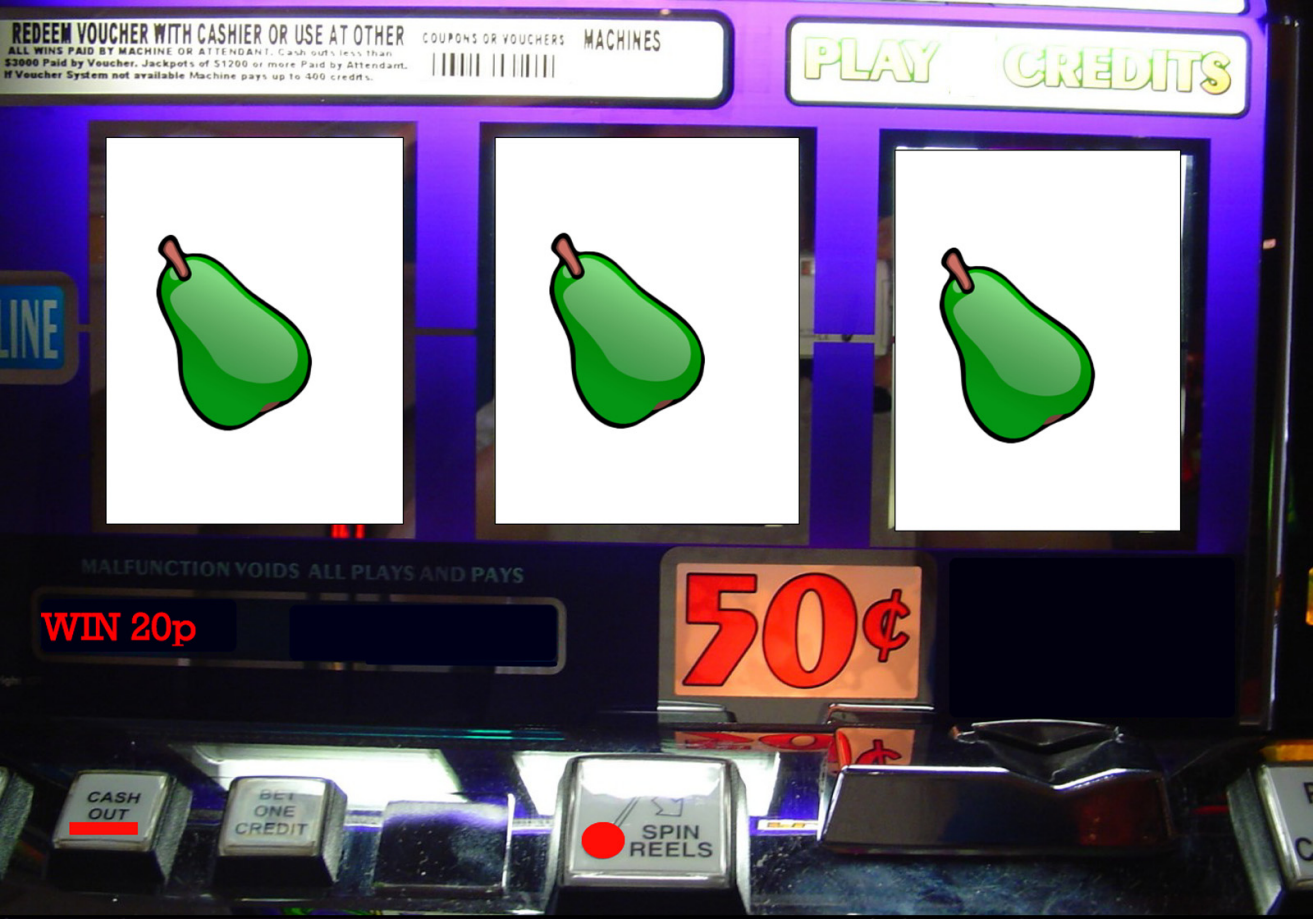
**Screenshot of the slot machine display participants were given during the partial reinforcement task**.

Participants were randomly assigned to one of four conditions. For the first part of the experiment, participants were asked to participate in a PREE paradigm in the context of a simulated slot machine (**Figure 1**). Participants were told how the slot machine worked, and the magnitude of the payoff for each type of winning outcome. The simulated slot machine was a simple one-line slot machine with three reels. Participants won money if the icons on three reels matched. There were five different icons (lemon, cherry, pear, orange, and lucky seven), with winning values of 10, 15, 20, 25, and 30p. The likelihood of each winning outcome occurring was the same, so the mean winning outcome was 20p ($0.35).

For each trial, participants were given the choice between gambling and skipping. The buttons were highlighted so that participants were aware of the two choices they had. Regardless of whether they chose to gamble or not, the images on the three reels presented on the screen refreshed every 500 ms to give the appearance of movement. At 1500, 3000, and 4500 ms, one of the reels (from left to right) stopped reeling. If the reels matched and the participant gambled, the participants was awarded money correspondent to value of the icons on the reel. If the reels did not match, they lost the wager they had made, which was fixed at 3p (£0.03, equivalent to around US$0.05). Wins and losses were accompanied by visual and auditory feedback which differed for each outcome. These noises were different if the participants skipped the gamble. Throughout the task participants were informed of their current balance. Between each trial, the buttons on the screen remained red, signifying that the participants were unable to make another wager. The ITI for the short ITI condition was 3000 ms, and 10000 ms for the long ITI condition.

Participants were presented with 10 practice trials before the game began crediting or deducting money from the player. Participants were informed when the practice trials had ended. Once the experimental trials began, participants played until they reached criterion, set as having won than £10.00 (US$15.40) in the bank. Once participants reached criterion, they were exposed to 50 trials of extinction, where it was not possible to win any money from the slot machine, and then the task ended automatically. Extinction was measured by the suppression of their gambling behavior; participants were not informed of the extinction phase at the end of the experiment. The practice trials had winning trials (which did not pay out), and the extinction phase had no wins or money. The practice and extinction phases were identical in each condition, bar the different ITI’s participants were exposed to.

After completing the PREE paradigm, participants were asked to make a series of contingency judgments about the effectiveness of a fictitious experimental drug related to patient recovery. The contingency judgment paradigm was adapted from a previously published study (Orgaz et al., 2013). In this paradigm participants were presented information about a fictional drug that was designed to cure a fictional infectious skin disease that had unpleasant consequences when an outbreak/crisis occurred. Participants were given the option of choosing between administering the drug and not administering the drug, and they were given feedback concerning the outcome immediately afterward (whether the patient’s situation had improved or not). The paradigm was designed to elicit illusions of control by having a high outcome density – the base rate of the desired outcome (patient recovered) was high (0.8), and was completely independent of the users decision. After making their decision, the participants were informed of the outcome of the choice, and there was a small pause (3500 ms) before being presented with the decision again.

After each set of 10 trials, participants were asked to judge the effectiveness of the drug. Participants were asked to judge the effectiveness of the drug on a scale from 0 to 100. This was represented by a shaded bar in the middle of the screen, on which they were given feedback about the number they chose, determined by how far along the bar they clicked. Participants could repeat clicking along the slider until they were happy with their choice, and were asked to confirm their choice using a separate button.

### Analytic Approach

To assess the length of extinction for each group, the proportion of gambles made were averaged across five blocks of 10 trials. Data analysis proceeded in two stages. Firstly, factorial ANOVAs were conducted on the extinction and contingency judgment data, with a 5 (block) × 2 (ITI) × 2 (Rate of Reinforcement) mixed design ANOVA being conducted. A 10 × 2 × 2 mixed design ANOVA was carried out on the 10 contingency judgments participants made. To test the effects of individual differences on gambling behavior and perseverative gambling, a series of poisson regression models were estimated on the number of trials participants gambled on during acquisition and extinction. This was conducted in three steps. First, an initial model was constructed where no covariates were entered into the model. Then, a second regression model was constructed in which ITI, rate of reinforcement, BIS scores, BDI scores and an interaction term between ITI and rate of reinforcement were included. ITI and rate of reinforcement were dummy coded (high ROR = 1, low = 0; short ITI = 1, long = 0), and BIS/BDI scores were rescaled with a mean of 0. This was compared against a null model using a likelihood ratio test (LRT). LRT’s are typically used in latent variable modeling to compare between two nested models, for example in latent class analysis (Collins and Lanza, 2010), or between the fit of two regression models, as in this case. This was then compared against a full model in which interaction terms were modeled across each covariate.

At this point, the data was tested to examine whether the data fit a poisson distribution. Crucially, poisson regression assumes that the conditional mean and variance are equal. While deviations from this assumption have little effect on the overall regression coefficients, when overdispersion (the variance being larger than the mean) is substantial this tends to depress standard errors, increasing the risk of false positive findings. While robust standard errors can be used to adjust these (Cameron and Trivedi, 2009), an alternative is to estimate a negative binomial regression model, which includes an extra parameter to model overdispersion. For the acquisition data, this approach was taken. For the extinction data, while the data was overdispersed the level of dispersion was considerably less, and so robust standard errors were applied to the regression model.

A number of outliers were found in the low rate of reinforcement extinction data. An examination of the data indicated that a number of gamblers in the low reinforcement, long ITI condition stopped gambling less than two gambles into extinction occurring and that these were outlying data points. These participants (*n* = 3) reported in debrief they treated £10 as salient, either stopping immediately after they won £10 or stopped to remain above £10, independent of any change in contingency. These participants were excluded from further analysis.

## Results

### Gambling Behavior

To study the effect of behavioral and trait variables on acquisition behavior, an offset negative binomial regression model was used to control for differential effects of exposure, where the same variables were used for the restricted and full factorial models as the extinction data. These revealed that the restricted model (**Table 1**) was a better fit than the null model (*G*^2^ = 22.74, *p* < 0.001), but that a full factorial model was no better fit than the restricted model (*G^2^* = 6.359, *p* = 0.784). This revealed that participants exposed to a higher rate of reinforcement gambled more frequently in acquisition.

**Table 1:** Offset negative binomial regression model of acquisition data.

Indicator	*B*	*SE*	*z*	*P*
Intercept	-0.224	0.031	-7.245	<0.001^∗∗∗^
ITI	-0.032	0.042	-0.758	0.448
ROR	0.122	0.046	2.689	0.007^∗∗^
BDI	0.001	0.002	1.097	0.273
BIS	0.000	0.002	0.059	0.953
ITI^∗^ROR	0.049	0.064	0.772	0.440


*^∗∗^*p* < 0.01, ^∗∗∗^*p* < 0.001.*

### PREE Task

**FIGURE 2:**
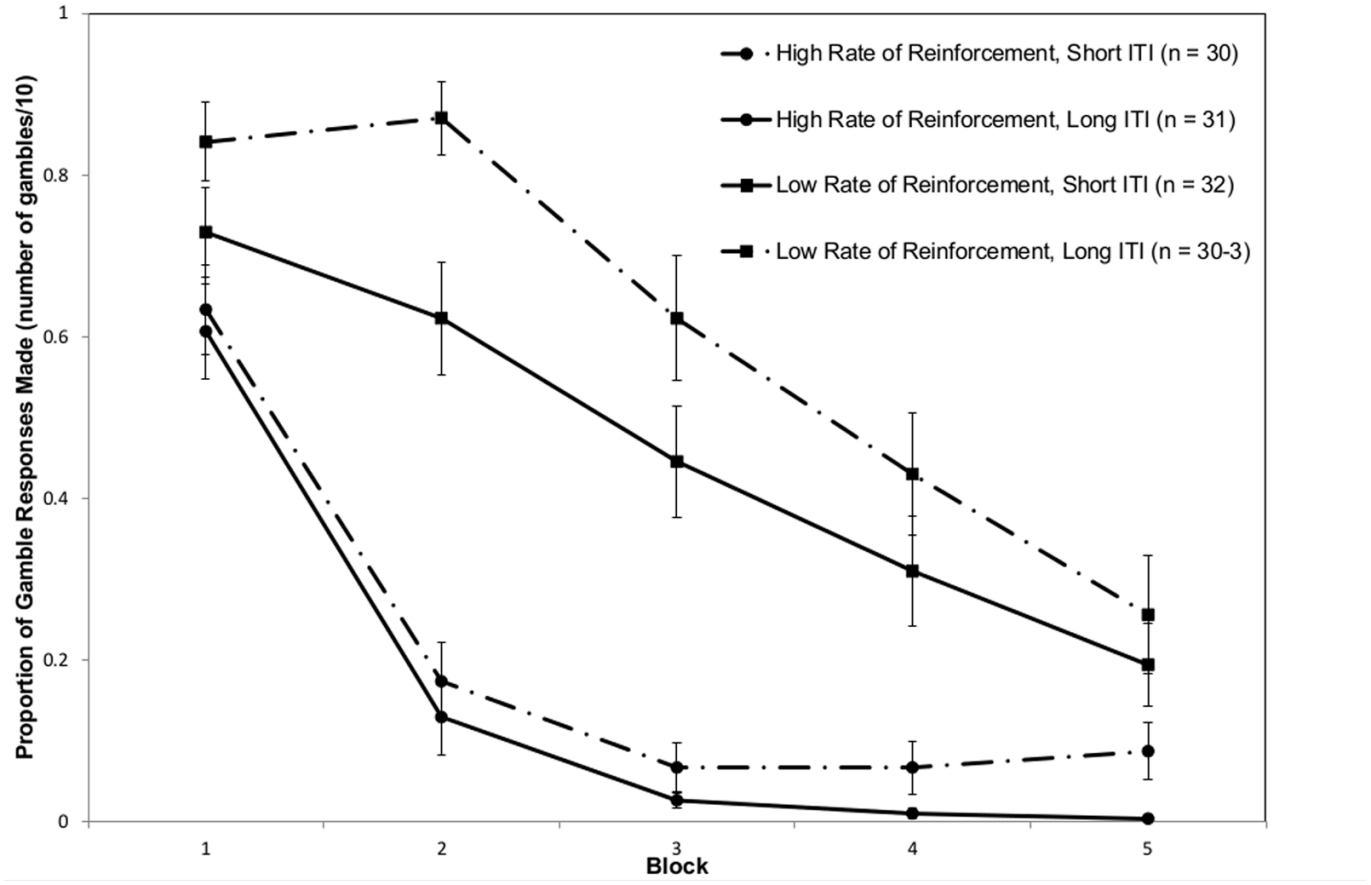
**Plot of extinction data for all groups, in blocks of 10 trials**.

The ANOVA conducted on the extinction data revealed main effects of block, *F*(2.541,292.187) = 131.095, *p* < 0.001, $\eta_{p}^{2}$ = 0.533, where the linear contrast was significant, *F*(1,115) = 229.457, *p* < 0.001, $\eta_{p}^{2}$ = 0.666, and the rate of reinforcement, *F*(1,115) = 82.912, *p* < 0.001, $\eta_{p}^{2}$ = 0.419, but no main effect of ITI, *F*(1,115) = 1.455, *p* = 0.23. There was an interaction between block and rate of reinforcement, *F*(2.541,292.187) = 22.801, *p* < 0.001, $\eta_{p}^{2}$ = 0.165, and a further interaction between the rate of reinforcement and ITI, *F*(1,115) = 6.317, *p* = 0.0133, $\eta_{p}^{2}$ = 0.052. There was no interaction between block and ITI, *F*(2.541,292.187) = 1.124, *p* = 0.334, or a three-way interaction, *F*(2.541,292.187) < 1. The main effect of block indicated that responses decreased as the block number increased (i.e., participants extinguished). This interacted with rate of reinforcement, as participants exposed to a higher rate of reinforcement extinguished more quickly, suggesting the presence of a PREE. The main effect of rate of reinforcement signified the same finding. The rate of reinforcement and ITI interaction indicated that when there was a low rate of reinforcement with a long ITI, participants gambled for longer in extinction (**Figure 2**). The block and rate of reinforcement effects, and the interaction between block and rate of reinforcement were all large in size ($\eta_{p}^{2}$ > 0.12), whereas the interaction between rate of reinforcement and ITI interaction was a small to medium effect.

### Individual Differences

**FIGURE 3:**
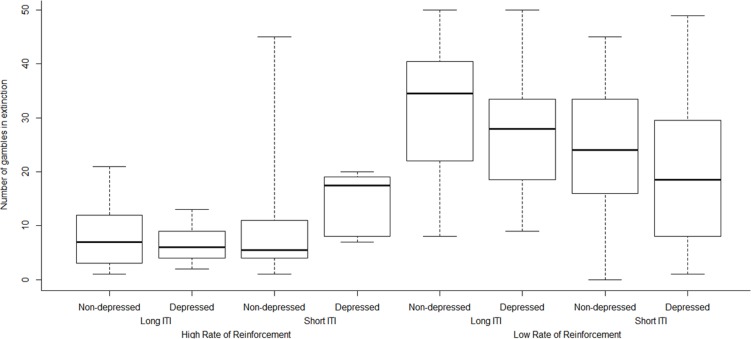
**Boxplot of depression status and proportion of gambles in extinction for each of the four conditions**.

To test the role of individual differences in perseverative gambling, a poisson regression procedure was used on the number of gambles in extinction The LRT indicated that the initial restricted model was a better fit of the data compared to the null model (*G*^2^ = 581.15, *p* < 0.001). The restricted regression model (**Table 2**) indicated that lower rates of reinforcement and longer ITI’s predicted longer perseverative gambling. These terms interacted in the same manner as the factorial ANOVA. A further regression model including interaction terms between the different covariates was subsequently conducted (**Table 3**) with the same variables as the regression in **Table 1.** A LRT comparing the restricted and full factorial regression models indicated that the full factorial model was a better fit of the data (*G*^2^ = 66.44, *p* < 0.001). This revealed the same significant effects as previously, but also that higher self-reported impulsivity predicted longer perseverative gambling. There was a trend suggesting that this interacted with rate of reinforcement, with less impulsive individuals appearing to persevere less in low reinforcement conditions. Scores on the two psychometric measures interacted, and there was a three way interaction between ITI, rate of reinforcement and BDI, with more depressed individuals in the high rate of reinforcement, short ITI group gambling for longer in extinction (**Figure 3**).

**Table 2:** Restricted poisson regression model of extinction data with robust standard errors.

Indicator	*b*	*SE*	*z*	*P*
Intercept	3.245	0.070	48.751	<0.001^∗∗∗^
ITI	-0.291	0.127	-2.303	0.021^∗^
ROR	-1.385	0.144	-9.587	<0.001^∗∗∗^
BDI	-0.010	0.004	0.931	0.134
BIS	0.004	0.006	-1.498	0.352
ITI ^∗^ ROR	0.565	0.239	2.366	<0.018^∗^


*^∗^p < 0.05, ^∗∗∗^p < 0.001.*

**Table 3:** Full poisson regression model of extinction data with robust standard errors.

Indicator	*b*	*SE*	*z*	*P*
Intercept	3.471	0.068	51.191	<0.001^∗∗∗^
ITI	-0.329	0.126	-2.620	0.009^∗∗^
ROR	-1.457	0.130	-11.208	<0.001^∗∗∗^
BDI	-0.011	0.008	-1.396	0.163
BIS	0.013	0.006	2.218	0.027^∗^
ITI ^∗^ ROR	0.620	0.237	2.617	0.009 ^∗∗^
ITI ^∗^ BDI	-0.012	0.016	-0.751	0.453
ROR ^∗^ BDI	-0.009	0.023	-0.383	0.701
ITI ^∗^ BIS	-0.011	0.011	-1.006	0.314
ROR ^∗^ BIS	-0.026	0.014	-1.911	0.056
BDI ^∗^ BIS	-0.002	0.001	-2.388	0.017^∗^
ITI ^∗^ ROR ^∗^ BDI	0.068	0.031	2.184	0.029^∗^
ITI ^∗^ ROR ^∗^ BIS	0.027	0.018	1.474	0.141
ITI ^∗^ BDI ^∗^ BIS	0.000	0.001	0.005	0.996
ROR ^∗^ BDI ^∗^ BIS	0.000	0.002	-0.058	0.954
ITI ^∗^ ROR ^∗^ BDI ^∗^ BIS	0.000	0.003	-0.037	0.971


*^∗^p < 0.05, ^∗∗^p < 0.01, ^∗∗∗^p < 0.001.*

### Contingency Judgment Task

**FIGURE 4:**
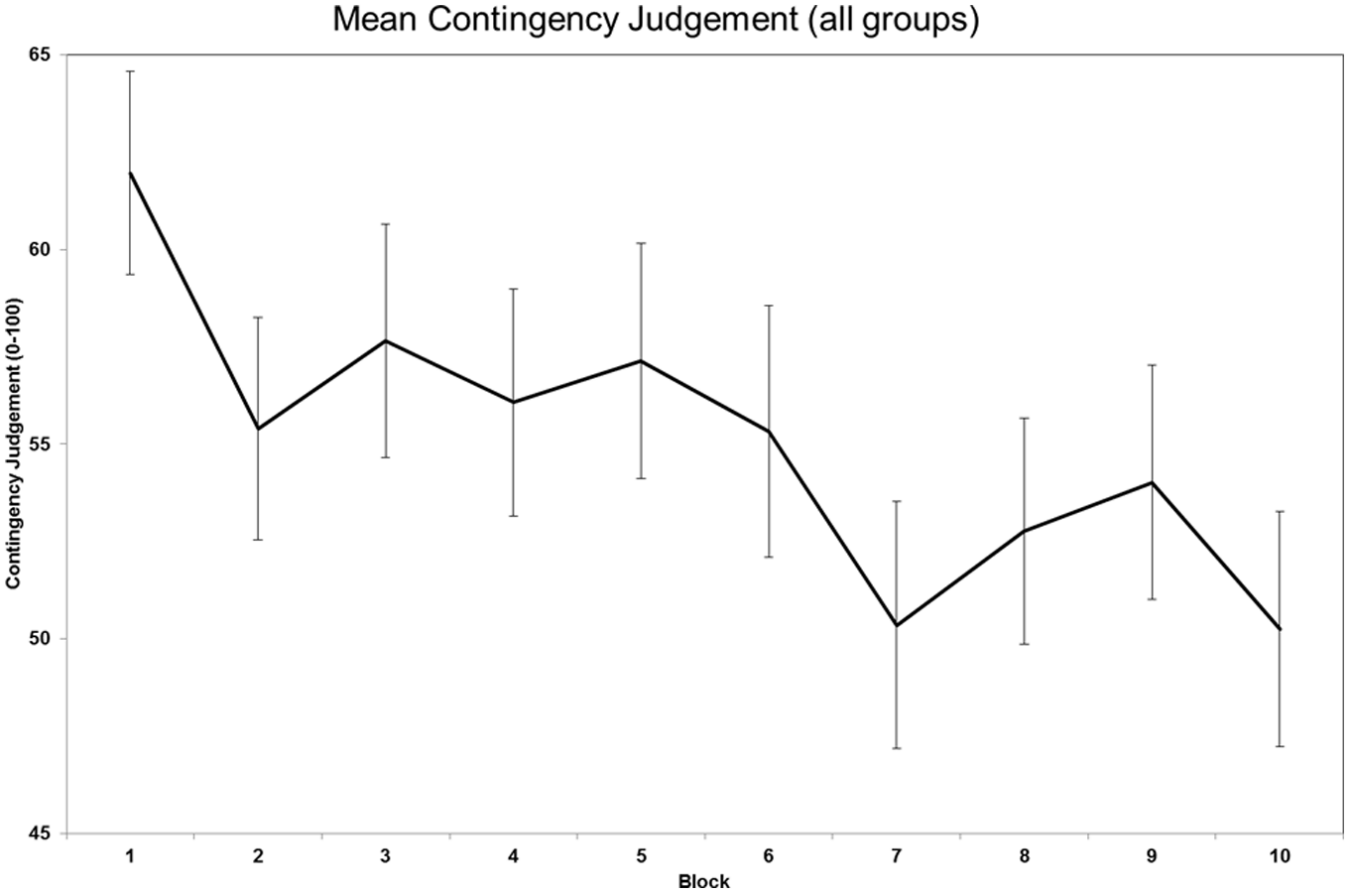
**Plot of mean contingency judgments across the 10 judgments participants made**.

Analysis of the contingency judgment data revealed that a significant main effect of block, *F*(6.526,737.416) = 3.735, *p* = 0.001, $\eta_{p}^{2}$ = 0.032. The main effect of block also included a significant linear contrast, *F*(1,113) = 10.312, *p* = 0.002, $\eta_{p}^{2}$ = 0.084, indicating that participants became better calibrated as they subsequently made judgments about the efficacy of the drug (**Figure 4**). Main effects of ITI, *F*(1,113) < 1, and rate of reinforcement, *F*(1,113) < 1, were not observed. Interactions between block and ITI, *F*(6.526, 737.415) < 1, block and rate of reinforcement, *F*(6.526,737.415) < 1, and ITI and rate of reinforcement, *F*(1,113) = 1.109, *p* = 0.295, were not significant. A three way interaction between block, rate of reinforcement, *F*(6.526,737.416) = 1.048, *p* = 0.399, was not significant either.

## Discussion

The results of this experiment demonstrate how different schedules of reinforcement affect behavior during a simulated gambling task, and can produce extended gambling in the face of continued losses. This also extends findings from a number of behavioral paradigms measuring perseverance to situations where participants are asked to name a specific preference. Both rate of reinforcement and ITI were instrumental in affecting how long participants gambled for when associations were extinguished, and these interacted. There was evidence that individual differences affected behavior under these conditions, with more impulsive individuals gambling for longer in extinction. In terms of rate of reinforcement, the findings of this study mirror an extensive literature that has repeatedly found that a leaner schedule of reinforcement is associated with greater perseverance in extinction. The findings concerning ITI (and the interaction term), have been predicted in the past, and a couple of studies have identified trial spacing effects in extinction with animals, but to our knowledge human research on this issue is very limited. This also highlights how the effects of timing on perseverative gambling have potential implications for gambling practice, particularly with newer gambling technologies being likely to alter the latencies between gambles. The impulsivity related findings speak to a literature that has previously suggested that impulsive individuals persevere for longer when the amount of money lost. This furthers research that highlights the importance of behavioral processes on gambling behavior, and has implications for gambling games and technologies, particularly those that encourage intermittent patterns of play.

Our findings broadly mirror a number of studies that used simulated slot machine paradigms to test partial reinforcement (Lewis and Duncan, 1956, 1957, 1958a,b; Poon and Halpern, 1971). We measured extinction slightly differently to previous studies, asking participants to choose whether to continue or not rather than when they walked away from the machine. Similar effects have been observed previously when asking people to choose between one of two machines (Dymond et al., 2012). It is important to note that it has been contested whether gamblers are able to discriminate between machines with different rates of reinforcement, measured in terms of preference (e.g., time spent on machine) between two or more simulated slot machines (Weatherly et al., 2004; Haw, 2008b; Dixon et al., 2013; Coates and Blaszczynski, 2014). We found that high rates of reinforcement were associated with a higher level of engagement on a simulated machine. This is broadly consistent with the literature, which has found that differences emerge but only when there is a sufficiently large enough gap in reinforcement. These results extend these to when different groups are exposed to different machines.

Both of the low reinforcement groups displayed extensive perseverative gambling. This continued gambling is potentially a behavioral marker of loss-chasing. Chasing losses is the often the first criterion of Disordered Gambling to emerge (Orford et al., 2010; Miller et al., 2013), and in models of problem gambling is theorized as a tipping point towards problem gambling. The extinction paradigm probes within-session continuation, a phenomena thought to be very closely related to loss-chasing in problematic gambling (Breen and Zuckerman, 1999). Partial reinforcement has previously been suggested as an alternative explanation for the phenomenon of loss-chasing (Dickerson, 1984), particularly for the continuation of gambling. Other explanations for loss-chasing tend to invoke the gamblers fallacy (Campbell-Meiklejohn et al., 2008). The results of this study provide support for the role of partial reinforcement in loss-chasing, albeit being limited to the perseverative aspects of chasing. Further research would need to be conducted on wager size to verify this. It should be noted though that in terms of clinical criteria (e.g., for Gambling Disorder in the DSM), there is a greater emphasis on perseverance. Similarly we found that impulsive individuals gambled for longer in extinction, a finding that has been previously observed in the literature (Breen and Zuckerman, 1999), and interpreted as demonstrating that impulsive individuals chase losses for longer.

Considering ITI, while we found that individuals persisted for longer in extinction with a longer ITI, their gambling behavior did not systematically differ in acquisition. The extinction finding appears to be somewhat more consistent with a trial based account of the PREE (Mackintosh, 1974), although we did not directly test between the two accounts. This finding somewhat contrasts with studies that have found that shorter latencies are associated with greater engagement (Linnet et al., 2010) and greater risk preferences (Hayden and Platt, 2007). We did not find that individuals preferred the longer ITI machines, but they did gamble for longer on them when forced to make a choice. A key qualification is that the development of slot machines indicates that machines have tended to speed up rather than slow down. However the way in which individuals interact with devices that can be used for gambling such as smartphones tends to increase latency, and is occasionally used within mobile video games for a similar purpose; players are offered the opportunity to gamble for an in-game valuable with large intervals (e.g., once a day), and can play again for real money. A similar concern is that some interventions aimed at reducing the harm caused by gambling intervene by forcing pauses within a gambling session. While this affects timing between sessions rather than trials, associative accounts of timing indicate a similar outcome. The findings of this study imply that care should be taken with these interventions. Moreover, this concern is not without empirical support, as a recent study has found that forcing breaks without including content to target gamblers’ attitudes or behaviors increases individuals’ motivations to continue gambling (Blaszczynski et al., 2015). Although this study explains these findings in the context of behavioral completion, an associative interpretation that is closely aligned with the present findings can be postulated.

The main effect of block (and a significant linear contrast) showed that participants’ gambling behavior was suppressed as extinction proceeded, and that extinction continued the longer that participants continued to lose. A main effect of rate of reinforcement was found. This is the classic PREE effect that has been observed in many studies since Humphreys (1939). These two main effects also interacted; behaviorally this is a restatement of the PREE, as the speed at which participants extinguished was faster with a high rate of reinforcement.

An interaction between the rate of reinforcement and ITI was also observed. The analyses strongly suggest that this interaction was driven by the low reinforcement, long ITI group, which appeared to show a resistance to extinction in the first two blocks (although no interaction with block was observed). Moody et al. (2006) found a similar pattern of results manipulating ITI in a partial reinforcement paradigm, albeit with much larger gaps between trials. This finding also appears to be consistent with Mackintosh’s (1974) review of extinction. This finding is particularly interesting in the context of newer gambling technologies, such as smartphone gambling, where larger gaps between gambles are anticipated because of how these devices are used. The Pathways Model (Blaszczynski and Nower, 2002), a well-supported model of problem gambling, predicts there are three pathways to problem gambling that share common associative learning and cognitive bases, and in particular that there is a ‘behaviorally conditioned pathway’ driven purely by this, compared to others which emphasize emotional vulnerabilities and antisocial/impulsive traits.

The only difference observed in the contingency judgment task was a main effect of block: participants’ judgments became better calibrated as the task progressed. The linear contrast on this was also significant, confirming the direction of the finding. Participants showed an illusion of control, as contingency judgments were substantially greater than relationship between response and outcome. There were no effects of ITI and rate of reinforcement. Given the unclear causal mechanisms underlying illusions of control (Orgaz et al., 2013), it might be that a behavioral processing deficit poses a risk factor for problem gambling. Consequently it would be interesting to examine whether performance on this task, taken prior to a gambling task, subsequently predicts gambling behavior.

We found that depressed individuals gambled for longer in the highly reinforced, short ITI group. Depressed individuals often prefer rapid, random games (e.g., slot machines) that produce negative reinforcement from poor mood (Blaszczynski and Nower, 2002). Problem gambling theories emphasize the importance of negative reinforcement in individuals experiencing traumatic life events or disordered mood; negative reinforcement is strongly hypothesized to be an important component in dependence related behaviours. With regard to ITI, resistance to expectancy changes observed in depressed and individuals (Abramson et al., 1978), in conjunction with changes in learning in depression due to ITI that has been used to explain the depressive realism effect might explain this finding. Specifically, the ITI and illusion of control literature identified that in positive contingencies, increases in ITI did not affect contingency judgment, but in depressed individuals these were inhibited in the same manner as non-contingent associations (Msetfi et al., 2005, 2007; Baker et al., 2010). Given that this line of research strongly suggests that ITIs affect behavior different in depressed people, it might be the case that increasing ITI has the same effect on expectancy changes as it does on contingency judgments, which might explain these findings. However this is speculative, and would require further research to investigated.

This study highlights how different schedules of reinforcement affect gambling behavior. Participants exposed to a lower rate of reinforcement persevered for longer. This interacted with ITI, as participants exposed to a longer ITI and a low rate of reinforcement gambled for longer in extinction. Participants with higher self-reported impulsivity gambled for longer in extinction. The results demonstrate that manipulating behavioral features in a simulated gambling game can produce longer perseverative gambling.

## Author Contributions

All authors listed, have made substantial, direct and intellectual contribution to the work, and approved it for publication. Richard James was responsible for the data collection and analysis. This work forms part of his doctoral research.

## Conflict of Interest Statement

The authors declare that the research was conducted in the absence of any commercial or financial relationships that could be construed as a potential conflict of interest.

## Footnotes

1 This study reports that their sample of high-frequency gamblers (*n* = 19) contained only three pathological gamblers, and the mean number of DSM-IV Pathological Gambling criteria endorsed was 2.3, indicating this is a difference found in low to moderate levels of problematic gambling.

2 A number of participants across conditions dropped out (*n* = 18). Participants who withdrew were resampled. All the participants who dropped out completed measures of depression and impulsivity. The majority of these dropouts (82%) were in the low rate of reinforcement, high ITI condition. Non-parametric tests were carried out to test whether the participants who dropped out differed from other participants from the same condition in any regard. No significant differences were observed in impulsivity or depression scores, nor the rate they were gambling prior to dropping out (Wilcoxon’s signed rank test, *p* > 0.05). All participants were debriefed upon withdrawal from the experiment. Participants who dropped out reported that they withdrew from the experiment because the length of the study conflicted with other engagements (e.g., lectures).
